# Enhanced CO_2_ Fixation Through Continuous Cultivation of Microalgae in a Two-Stage Photobioreactor System

**DOI:** 10.3390/molecules31152627

**Published:** 2026-07-28

**Authors:** João Tavares, Susana M. Paixão, Tiago P. Silva, Luís Alves

**Affiliations:** Unidade de Bioenergia e Biorrefinarias, LNEG—Laboratorio Nacional de Energia e Geologia I.P., Estrada do Paço do Lumiar 22, 1649-038 Lisboa, Portugal

**Keywords:** two-stage photobioreactor, continuous culture, nutrient uptake, CO_2_ fixation, microalgae biomass, circular bioeconomy

## Abstract

The integration of microalgae cultivation into biorefinery systems represents a promising strategy to enhance carbon dioxide (CO_2_) sequestration and support sustainable biomass production for diverse biotechnological applications. This study reports the CO_2_ fixation performance and biomass productivity of a two-stage photobioreactor (PBR) system employed for the continuous cultivation of *Haematococcus pluvialis*, utilizing biogenic CO_2_ generated from a heterotrophic culture. The first stage (ST1) operated as an autotrophic chemostat (ST1 PBR) with variable dilution rates (0.18–1.00 d^−1^), while the second stage (ST2) consisted of sequential high-irradiance columns that promoted biomass and carotenoid accumulation. The two-stage PBR (ST1 + ST2 PBR) achieved a maximum CO_2_ assimilation capacity of 0.747g/L/d and up to 94% CO_2_ fixation efficiency. The daily CO_2_ assimilation rate reached 6.53 g/d, representing a 10% improvement compared with the single-stage system (ST1 PBR). The maximum overall biomass productivity reached 0.332 g/L/d when both PBR stages were operated together, whereas the second stage alone achieved up to 1.18 g/L/d under continuous operation and a maximum biomass concentration of 3.33 g/L during batch-induced carotenogenesis. Nutrient uptake analysis revealed an increasing fraction of unconsumed major nutrients with higher dilution rates. Continuous operation under high irradiance did not induce pigment accumulation, whereas batch mode successfully triggered carotenogenesis over 13 days, resulting in visible pigment production and high biomass yield. Overall, the two-stage PBR system demonstrated high CO_2_ capture efficiency, enhanced biomass productivity, and operational flexibility, offering a scalable and sustainable approach for integrating microalgal cultivation into biorefineries while contributing to carbon mitigation and circular bioeconomy goals.

## 1. Introduction

Microalgae are autotrophic microorganisms found in marine and freshwater environments, capable of converting sunlight into chemical energy through photosynthesis. Through this process, they can capture atmospheric CO_2_ and produce a variety of valuable biologically active substances, ranging from simple sugars to polysaccharides, proteins, lipids, pigments, enzymes, etc. Thus, microalgae cultivation has attracted widespread attention both for its ability to fix CO_2_ at rates 10 to 50 times higher than those of terrestrial plants [1] and for its capacity to convert the captured carbon into valuable biomass, which can be utilized as a feedstock for food, animal feed, bioenergy, and a wide range of bioproducts [2].

The biotechnological potential of microalgae has long been recognized [3], and their incredible diversity, combined with their ability to thrive and rapidly proliferate in harsh environmental conditions, makes them promising candidates for industrial exploitation. Early research on microalgae primarily focused on mass cultivation and strain selection, driven by the search for alternative sources of unconventional proteins, lipids, essential nutrients, and vitamins [3,4,5]. Subsequently, interest shifted toward the discovery of bioactive compounds and valuable metabolites, such as pigments (astaxanthin, β-carotene), polyunsaturated fatty acids, and vitamins, mainly for nutraceutical and pharmaceutical applications [5,6]. Over the last three decades, intensive research has targeted microalgal biofuels, including biodiesel, bioethanol, biogas, and biohydrogen, with efforts directed toward enhancing lipid productivity, applying genetic engineering, and reducing costs in large-scale systems [7,8,9,10]. Over the last two decades, the focus has shifted toward the use of microalgae for carbon sequestration and wastewater remediation, with particular attention to industrial flue gas capture and nutrient recovery from wastewater [11,12], and the advancement of integrated microalgal biorefineries aligned with circular economy principles [13,14].

All these applications and exploitation of microalgae can indeed benefit from the use of industrial CO_2_ streams, including combustion flue gases, syngas, biogas, fermentation off-gases, gases from wastewater aeration, and composting off-gases. However, when targeting high-value purposes, such as human food, cosmetics, or pharmaceuticals, the origin and composition of the CO_2_ streams must strictly comply with established safety and quality standards [15,16,17]. In such cases, biogenic CO_2_ streams offer greater potential because they are more likely to meet safety and quality requirements, since the concentration of substances that could compromise product quality or pose risks to human health is typically low to null [18].

Industrial CO_2_ capture through microalgae cultivation can be carried out using traditional open systems (e.g., open ponds, raceway ponds) or closed systems (photobioreactors). While open cultivation systems offer advantages such as ease of scale-up, simple operation, and low operational costs, they suffer from significant limitations. Due to the low solubility of CO_2_ in water (≈approximately 1.45 g/L at 25 °C and 1 atm), a significant fraction of the CO_2_ supplied to the culture escapes into the atmosphere, resulting in a low CO_2_ mass-transfer efficiency [19]. Additionally, open systems face challenges such as high-water evaporation and limited control over cultivation conditions. In closed systems, despite the complexity and high capital and operational costs, the microalgae cultivation conditions can be controlled as close as possible to the optimal. The CO_2_ residence time, the gas–liquid mixing, and the interphase contact area can be significantly improved to boost the CO_2_ mass-transfer, allowing for better productivity [20], thereby reducing overall photobioreactors cultivation costs.

Reactor design is thus fundamental to maximize efficiency and lead the process towards the intended goals. In this context, Tavares et al. [21] have developed a novel photobioreactor (PBR) design that allows for continuous cultivation of photoautotrophic microalgae under chemostat conditions, using a continuous stream of CO_2_-rich gas as the sole carbon source. Compared with conventional photobioreactors, the novel modular recirculating design improves culture homogenization, light distribution, and gas–liquid mass transfer, enabling stable long-term continuous cultivation under controlled conditions. Furthermore, the recirculation system allows the different components to act as single chemostat and not as a series of sequential individual components, ensuring the production of stable and homogeneous cultures, that respond proportionally to different stimuli. This system also allows independent variation in aeration, feeding and recirculation rates which is fundamental when studying the factors that influence growth rates, biomass concentration a carbon fixation.

The reactor was operated using *Haematococcus pluvialis* as an industrial model microalgal strain. This microalga was selected since it is well-known for its autotrophic properties and application on a commercial scale, particularly as a source for natural astaxanthin [22,23,24,25]. This initial design was successful in maintaining stable cultures for long periods of time and provided proportional responses to different stimuli. Nonetheless, it resulted in relatively low carbon capture rates and was not ideal for carotenoid production.

So, this study aims to address the highlighted issues in two different ways. First, by improving on the previous PBR design (chemostat), reaching a configuration 1 designated as stage 1 (ST1) and, second, by incorporating an additional sequential multi-column module to achieve greater CO_2_ sequestration and carotenoid production, designated as stage 2 (ST2), reaching a combined configuration 2. The proposed system offers a promising strategy for the simultaneous capture of biogenic CO_2_ and production of microalgal biomass, supporting the development of more sustainable and circular biorefinery processes.

In this context, *Haematococcus pluvialis* was cultivated in the improved PBR, serving as a continuous microalgal CO_2_ capture module integrated into a biorefinery system, which comprised a continuous production of biodesulfurization biocatalysts via heterotrophic cultivation of *Gordonia alkanivorans* strain 1B [26] and a continuous bio-oil biodesulfurization process [27]. The microalgal CO_2_ capture module continuously captures and utilizes the CO_2_ released during biocatalysts cultivation and biodesulfurization, thereby lowering direct emissions.

**Figure 1:**
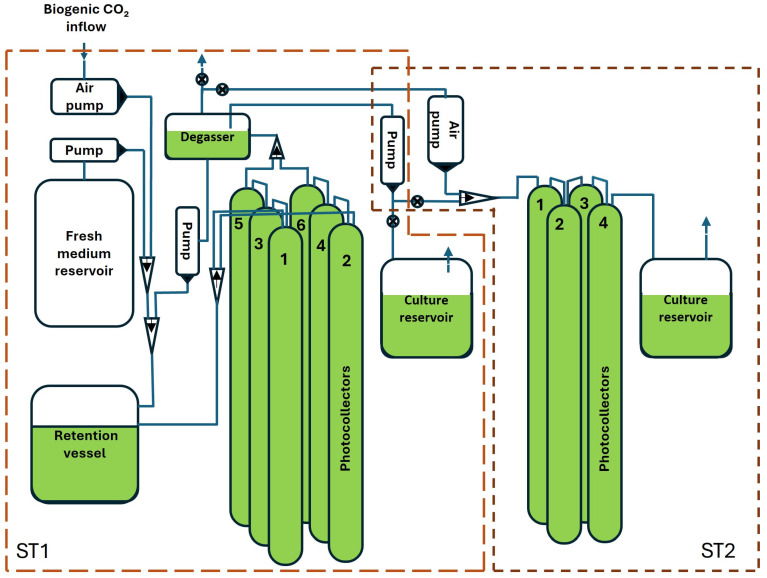
Schematic diagram of the benchtop PBR system used in this study (not to scale). Configuration 1 corresponds to an updated version of the photobioreactor developed by Tavares et al. [21] and includes only stage 1 (ST1 PBR), whereas Configuration 2 incorporates both stages (ST1 + ST2 PBR). The column numbering in ST1 reflects the culture flow direction in two parallel paths (1–3–5 and 2–4–6). In ST2, the columns are numbered sequentially from 1 to 4, following the culture flow direction. Arrows indicate the direction of fluid and gas flows, while triangles denote points of convergence or division of flows. The symbol (circle with an internal cross) represents a check valve. The biogenic CO_2_ flow originates from an aerobic heterotrophic chemostat [27].

Thus, to demonstrate the effectiveness of the design changes and evaluate the performance of CO_2_ capture and biomass productivities in the upgraded PBR system (see Figure 1), *H. pluvialis* cells were first cultivated in the ST1 configuration (configuration 1) under different dilution rates, ranging from 0.18 d^−1^ to 1 d^−1^. This allowed for the evaluation how growth rate influences CO_2_ absorption, nutrient uptake, and overall productivity. Afterwards, ST2 was coupled to the PBR system (configuration 2) and tested in sequential continuous cultivation and continuous gas feeding to maximize CO_2_ absorption and additional biomass accumulation. For all tested dilution rates, biomass productivity and CO_2_ consumption were systematically analyzed, enabling a comprehensive evaluation of the overall upgraded PBR system’s efficiency. In addition, ST2 was also tested in batch cultivation with continuous gas feeding to promote induction of carotenogenesis. The whole ST1 + ST2 PBR system constitutes the configuration 2.

## 2. Materials and Methods

### 2.1. Microorganism and Culture Medium

The seed culture of *Haematococcus pluvialis* Flotow used in this work was kindly provided by ALISU—Coleção de Algas da Universidade de Lisboa (Faculty of Sciences—Lisbon University, Lisbon, Portugal). Stock cultures were grown and maintained in synthetic Bold’s Basal Medium (BBM), without vitamin supplementation, as described by Tavares et al. [21]. BBM was consistently used in all PBR experimental assays.

### 2.2. Biogenic CO_2_ Stream

The CO_2_ supplied in the experiments was produced by a continuous bacterial culture (*Gordonia alkanivorans* strain 1B), cultivated as described by Tavares et al. [28]. To sustain bacterial growth, atmospheric air was continuously introduced into the aerobic chemostat, resulting in the production of exhaust gases at a stable rate of 1.3 L/min. These gases, characterized by an average composition of 0.29 ± 0.04 vol% CO_2_ and 20.25 ± 0.22 vol% O_2_ (other atmospheric components were not analysed), were subsequently supplied to the photobioreactor (PBR), serving as the sole carbon source for the system.

### 2.3. Photobioreactor Assembly and Operation

The photobioreactor employed in this work was a closed PBR system based on the design developed by Tavares et al. [21], operated in two different configurations (configuration 1 = ST1 PBR and configuration 2 = ST1 + ST2 PBR) as presented in Figure 1.

#### 2.3.1. Configuration 1

Configuration 1 (ST1 PBR) was an improved version of the previous PBR design. It maintained the three main elements that characterized the design by Tavares et al. [21], namely the retention vessel, a photocollector unit, composed of a series of glass columns, and the innovative recirculation unit that allowed the system to operate as a single photoautotrophic chemostat.

What sets the ST1 PBR system apart from the previous design are the changes added to the photocollector unit. In this modified design, the columns were arranged into two parallel sets, each consisting of three columns in series. The circulation of the culture through the columns was optimized to ensure complete column filling, which increased the total working volume from 6.0 ± 0.2 L [21] to 7.9 ± 0.2 L. This volume increase in the photocollector unit pretends to result in greater light exposure and overall greater photosynthetic performance. The overall rearrangement also allowed the system to be operated at greater pressures and with greater flow rates for both gas and liquid.

In this first set of experiments, the PBR was operated, in a single-stage (ST1, Figure 1), in continuous mode as an autotrophic chemostat. Following sterilization and assembly of the PBR system under aseptic conditions [21], 7 L of autoclaved BBM were introduced into the reactor, followed by the addition of 0.9 L of the microalgal inoculum. The culture was initially grown in batch mode, for 3 days, until reaching the exponential growth phase, under the following conditions: an internal recirculation rate of 0.67 L/min, an input air flow of 1.3 L/min (0.165 vvm), a temperature of 21 ± 1 °C, a pH of 7.5 ± 0.2, and an irradiance of approximately 50 μmol.m^−2^·s^−1^ at the vertical column surface (see Section 2.4). Thereafter, the PBR was supplied with sterile BBM in a continuous flow, applying dilution rates between 0.18 and 1 d^−1^. These operating conditions corresponded to hydraulic residence times (HRT) varying from approximately 133 h to 24 h, respectively. At each dilution rate, the operating conditions were sustained for a period sufficient to complete at least three full turnovers of the total working volume, as determined by the dilution rate (t ≥ (1/D) × 3; t = time; D = dilution rate), ensuring system stability and the establishment of steady-state conditions in the chemostat. During the experiments, growth parameters were regularly monitored, and both inlet and outlet gases were analysed daily. For each steady-state condition, analyses were performed using at least four analytical replicates obtained from a minimum of two independent culture samples.

#### 2.3.2. Configuration 2

Configuration 2 further improves on the original design by Tavares et al. [21] and combines the initial single-stage (stage 1—ST1) system with an additional high irradiance photocollector unit, consisting of an array of four sequential columns (stage 2—ST2), which adds 3.1 ± 0.1 L to the total working volume (Figure 1, ST1 + ST2).

Unlike the three initial units that composed ST1, the high irradiance photocollector unit does not operate as an integrated part of the autotrophic chemostat, but instead as a sequential addon component, resulting in a two-stage operating system. This unit was devised to enhance the fixation of residual CO_2_ from the bacterial chemostat that could not be fixed in ST1.

In configuration 2 (ST1 + ST2 PBR), the outflow gas stream, resulting from ST1 is transferred to ST2, using an air compressor, regulated by a precision rotameter. This allows the system to maintain a continuous air flow, without significant pressure fluctuations, by matching the air inflow from the heterotrophic bioreactor to ST1, and the air outflow from ST1 to ST2. Air is transferred sequentially from the headspace of each column to the bottom liquid phase of next. The air stream serves as a carbon source while simultaneously ensuring homogenization, through the bubbling effect.

In terms of irradiation, ST2 is continuously illuminated (24 h photoperiod) by two sets of four fluorescent lamps (Philips T8, 60 cm, 18W/865, 6500 K, Amsterdam, The Netherlands), each set delivering approximately 4000 lumens to one side. The irradiance measured at the vertical surface of the columns was approximately 200 μmol·m^−2^·s^−1^.

ST2 was operated in two different modes: (1) In continuous mode—in which a peristaltic pump continuously transfers the microalgal culture, collected from the culture outflow at the degasser vessel in the ST1 to the first column of ST2. In this mode, both air and liquid are transferred from the top part of each column to the bottom part of the next. The airflow serves as a driving force to promote the transfer of cells between columns and both air and liquid flow to promote mixing. This results in a continuous culture, but not a chemostat, as cells are continuously being fed to the systems, being at different stages throughout the different columns. After exiting the last column air and cells are separated, and then cells are collected in an appropriate vessel. This setup was selected to promote both residual CO_2_ fixation and biomass production. During operation, five different dilution rates were tested 0.18–1.0 d^−1^. These dilution rates were determined by the rate of ST1, as transfer between systems was always constant. (2) In batch mode—in which there is continuous feeding with air from ST1, but no feeding of liquid. In this mode, there is no active transfer of culture between columns, which coupled with the high irradiation and abundant carbon available in the air stream, may lead to nutrient starvation, which can induce microalgae carotenogenesis.

In this set of assays, after sterilization and assembly in aseptic conditions, ST2 was inoculated with *H. pluvialis* culture drawn from the ST1 and maintained under intense irradiance (≈200 μmol·m^−2^·s^−1^) for 13 days to stimulate carotenoid accumulation.

### 2.4. Analytical Methods and Kinetic Parameters

Microalgal growth was evaluated through two parameters: optical density at 600 nm (OD_600_), using a Thermo Scientific Genesys 20 spectrophotometer (Thermo Fisher Scientific Inc., Waltham, MA, USA), and dry cell weight (DCW, g/L), both measured at steady-state conditions. To determine DCW, samples were collected overnight to a closed flask placed on an ice bath, preventing further proliferation, followed by filtration of 100 mL aliquots through 0.22 μm fiberglass filters (Millipore, Burlington, MA, USA). The filters were then dried at 105 °C for 24 h and weighed to obtain biomass concentration. Each steady-state measurement was carried out in at least two independent replicates.

Incident light on the vertical surface of the photocollector columns was quantified using a TES-1330 digital light meter (TES Electrical Electronic Corp., Taipei, Taiwan). Gas composition at the PBR inlet and outlet, specifically CO_2_ and O_2_ contents (vol%), was analyzed with a Servomex 1440 analyzer (Crowborough, East Sussex, UK). The carbon fraction (C%) in the microalgal biomass was measured by elemental analysis following ISO 16967:2015 [29].

Soluble inorganic anions, including chloride, nitrate, phosphate, and sulfate, were analysed by the Laboratory of Biofuels and Biomass (LBB), an accredited analytical laboratory at LNEG, using suppressed ion chromatography [30]. The concentrations of calcium, magnesium, sodium, and potassium were determined, by the LBB, via flame atomic absorption spectrometry [31,32].

All kinetic parameters were calculated using the standard equations described by Tavares et al. [21]. For each steady-state, the following parameters were evaluated: daily biomass productivity (Px), volumetric productivity (Pv), CO_2_ assimilation capacity (AC), O_2_ production capacity (PC), specific rates of CO_2_ fixation (A_CO2_) and O_2_ release (A_O2_), carbon fixation efficiency (CFE, %), and yield of CO_2_ incorporation into biomass (Y).

For all experiments, the standard uncertainty (u) was evaluated. A mass uncertainty, u(m), of 0.1 mg was assigned to each weighing, and a temperature uncertainty, u(T), of 0.5 °C was assumed for all measurements. Experimental errors depended on the calibration method, and the errors of measured values were calculated as the standard deviation. Each measurement was performed at least in triplicate, and results are reported as mean ± standard deviation. Descriptive statistical analyses were conducted using the Data Analysis tool in Microsoft Excel.

## 3. Results and Discussion

### 3.1. Chemostat—Configuration 1

#### 3.1.1. Growth Kinetic Parameters

**Figure 2:**
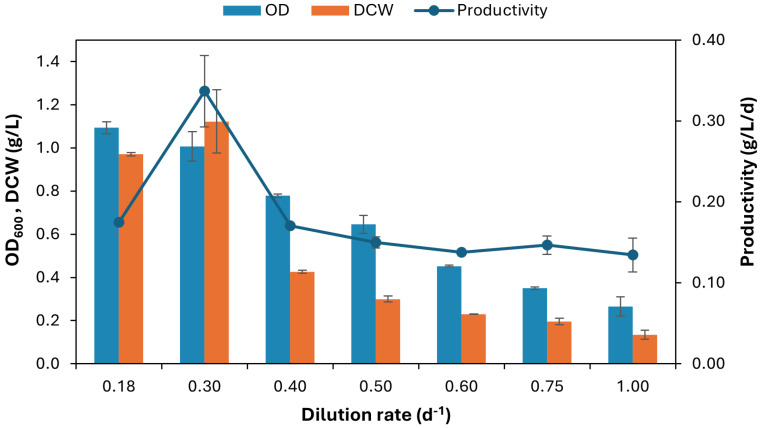
Biomass concentration (DCW), optical density (OD_600_), and volumetric productivity (Pv) of *H. pluvialis* in a continuous chemostat PBR (ST1 PBR) as a function of increasing dilution rates (0.18–1.0 d^−1^). Error bars indicate the standard deviation of triplicate measurements (*n* = 3) at each steady-state.

To assess the impact of the PBR design improvements on CO_2_ capture efficiency and biomass productivity, the upgraded PBR system in its first configuration (ST1 PBR) was operated as a continuous autotrophic chemostat culture of *H. pluvialis*, under seven different dilution rates ranging from 0.18 to 1.00 d^−1^. Figure 2 presents the variation in the culture optical density (OD_600_), dry cell weight (DCW), and volumetric productivity (Pv) as a function of the dilution rates. The main kinetic parameters of the chemostat culture are also provided in Table 1. As can be observed, OD_600_ decreased as the dilution rate increased. Between dilution rates of 0.18 and 0.3, the reduction was minimal (8%). However, from 0.3 to 1.0, the decrease became more pronounced, ranging from 17% to 30% between consecutive dilution rates. DCW increased from 0.97 g/L at a dilution rate of 0.18 d^−1^ to 1.12 g/L at 0.3 d^−1^. Beyond this point, biomass concentration dropped sharply (62%) at the next dilution rate and continued to decline progressively across all subsequent dilution rates up to 1 d^−1^. A similar trend was observed for volumetric productivity, which peaked at 0.34 g/L/d at a dilution rate of 0.3 d^−1^, representing nearly twice the maximum volumetric productivity of 0.183 g/L/d achieved in our previous work at the same 0.3 d^−1^ dilution rate [21]. Thereafter, productivity declined sharply (49%) at a dilution rate of 0.4 d^−1^ and then stabilized at approximately 0.14 g/L/d across all subsequent dilution rates up to 1 d^−1^ (Figure 2).

It is well established that *H. pluvialis* proliferates through a complex asexual life cycle [33], with maximum vegetative biomass generally occurring near the optimal growth rate in autotrophic cultivations. In this study, however, when exposed to dilution rates exceeding the typical 0.3–0.4 d^−1^ growth rate reported for autotrophic cultivation [34,35,36], the cultures were still able to sustain steady states, maintaining productivity at a plateau across dilution rates up to 1 d^−1^, although the biomass concentration progressively decreased at each steady-state. This behaviour contrasts with the expected outcome, where such high dilution rates would typically lead to rapid washout. This suggests that at dilution rates close to the optimal growth rate, *H. pluvialis* efficiently utilizes nutrients from the medium and assimilated CO_2_ to maximize biomass accumulation. In contrast, when exposed to dilution rates above those values, the culture undergoes fast-growing population selection, allowing cells to adapt and sustain growth at a rate matching the applied dilution rate. However, it is important to recognize that the growth rate is inherently influenced by multiple cultivation factors, including the culture medium, cultivation mode, light intensity and quality, the type and design of the photobioreactor and its mixing efficiency, the concentration of supplied CO_2_, among others [36,37,38]. Some high specific growth rates have been reported for *H. pluvialis* when some factors are exploited to the extreme. In a study from our team in 2023 [21], using a continuous chemostat photobioreactor, it was shown that *H. pluvialis* can tolerate dilution rates of up to 0.5 d^−1^ under typical temperature and illumination conditions, although without productivity increase beyond a dilution rate of 0.3 d^−1^. A specific growth rate of 0.49 d^−1^ was reported by Wu et al. [39], achieved through enhanced carbon mass transfer using microbubbles with a continuous 1% CO_2_ supply. Pang et al. [40] cultivated *H. pluvialis* under mixotrophic conditions using sodium gluconate (2 g/L) as an organic carbon source combined with high light intensity, achieving a specific growth rate of 0.48 d^−1^. Using a genetic transformation approach, Cheng et al. [41] achieved a specific growth rate of 0.60 d^−1^, representing a 15% increase over the wild-type strain, in a mutated *H. pluvialis* culture aerated with a 10% CO_2_ air stream, reaching a peak biomass productivity of 0.16 g/L/d. Numerous strategies have been explored to enhance the growth rate and productivity of *H. pluvialis*; however, to the best of our knowledge, studies involving continuous cultivation systems remain scarce. Fábregas et al. [42] developed an optimized culture medium for *H. pluvialis* and achieved a steady-state at a semi-continuous dilution rate of 0.2 d^−1^, resulting in cell density and productivity (75.5 × 10^6^ cells/L/d) that were three times higher than those obtained using the standard BBM culture medium. Another study, by García-Malea et al. [43], reported a maximum biomass productivity of 0.58 g/L/d at a dilution rate of 0.96 d^−1^, using a nitrate-rich medium (0.765 g/L NO_3_^−^) and high irradiance (2500 μE·m^−2^·s^−1^). Under these conditions, the culture reached a cell density of approximately 2.5 × 10^6^ cells/mL. In semi-continuous cultivation of *H. pluvialis* using a panel-type photobioreactor, Imamoglu et al. [23] reported a specific growth rate of 0.27 d^−1^ and a maximum cell density of 1.06 × 10^6^ cells/mL, corresponding to a productivity of 0.23 g/L/d. Similarly, Ranjbar et al. [44] achieved a high biomass productivity of 0.40 g/L/d in a bubble column photobioreactor, reaching a maximum cell density of 6 × 10^6^ cells/mL under semi-continuous operation.

**Figure 3:**
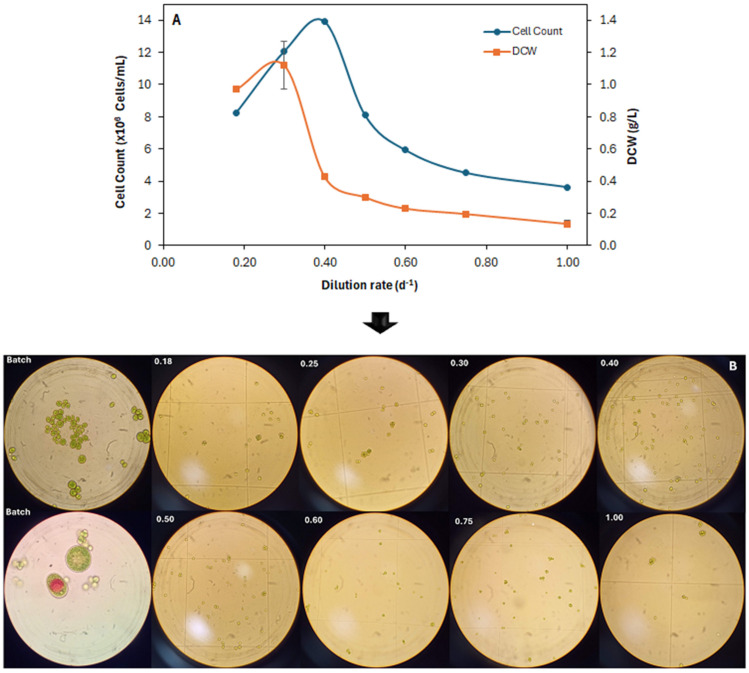
Growth behaviour and morphology of *H. pluvialis* in batch and continuous chemostat PBR at different dilution rates (0.18–1.0 d^−1^). The graph (**A**) shows cell density (×10^6^ Cells/mL) and biomass concentration (DCW) as a function of dilution rate, while the microscopic images (**B**) illustrate the cell morphology under batch and continuous cultivation conditions across the same dilution rate range. For batch conditions two images are presented to illustrate the diversity of cell morphology. Images were acquired using a Neubauer hemocytometer, in which each grid segment corresponds to 50 µm.

In the present study, as shown in Figure 3, the maximum cell density of 1.39 × 10^7^ cells/mL was achieved at 0.4 d^−1^ dilution rate, which was a rapid increase from the 8.21 × 10^6^ cells/mL observed at 0.18 d^−1^. For dilution rates above 0.4, the cell density decreased rapidly (42%) to the next rate and then progressively slower until the minimum of 3.6 × 10^6^ cells/mL at 1.0 d^−1^ dilution rate (Figure 3A). Microscopic observations (Figure 3B) revealed that the average cell diameter progressively decreased with increasing dilution rates. Until dilution rates up to 0.3 d^−1^, spherical green cells without flagella predominated. At higher dilution rates, a growing proportion of pear-shaped green cells with two flagella and some with a distinct gelatinous cell wall was observed, indicating a morphological shift likely associated with accelerated cell division and adaptation to faster growth conditions, in which cells eventually leave the chemostat before they have time to mature, leaving in the reactor a population of low-diversity cells. Notably, although cell number increased between dilution rates of 0.18 and 0.4 d^−1^, the biomass concentration dropped sharply between 0.3 and 0.4 d^−1^, suggesting a shift toward smaller and less dense cells at higher growth rates, as already observed by Tavares et al. [21]. These observations are consistent with established chemostat dynamics, where increasing dilution rates impose selective pressure favouring faster-growing cell phenotypes at the expense of population density and maturation. This supports the idea that under high dilution rates, *H. pluvialis* populations adapt morphologically to sustain growth, albeit as smaller, less mature forms, aligning with theoretical models of chemostat evolution [45].

#### 3.1.2. Nutrient Uptake

**Figure 4:**
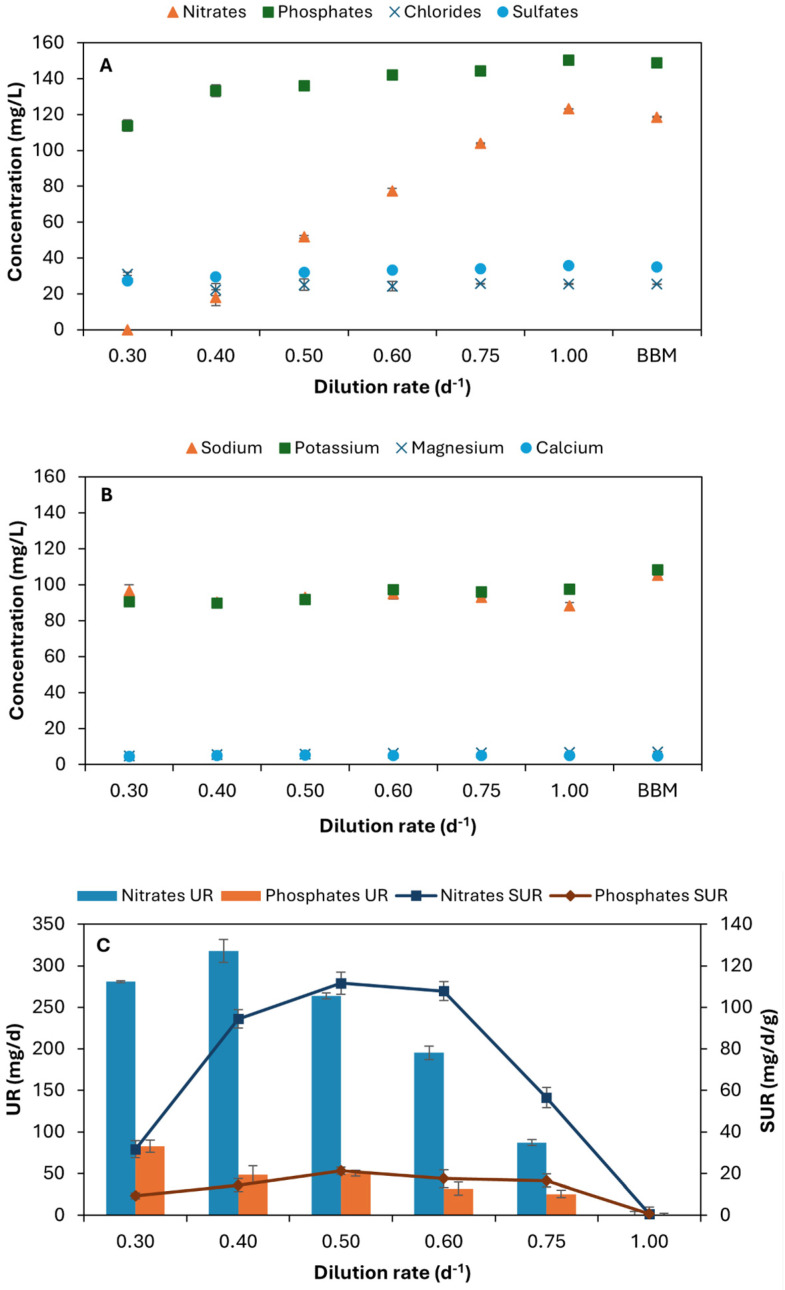
Residual nutrient profiles in the chemostat effluent at different dilution rates (0.30–1.00 d^−1^). Plots (**A**,**B**) show the main macronutrient remaining concentrations, in comparison with culture medium (BBM) initial composition, and plot (**C**) shows daily and specific uptake rates (UR = uptake rate; SUR = specific uptake rate) of the two principal macronutrients (N and P sources) by the microalgal biomass in the chemostat (ST1 PBR).

Chemostat PBR systems have the advantage of balancing nutrient supply through a continuous fresh medium supply, thereby avoiding limitations or excesses that can significantly affect cell division, microalgal growth, overall biomass composition, and the productivity of molecules of biotechnological interest [42,46,47,48]. Although continuous cultivation enables microalgae to grow close to their maximum or desired growth rate, the use of high dilution rates in non-optimised culture media can result in substantial nutrient losses, which not only represent an economic drawback but also increase downstream treatment costs [49]. Taking this into account, herein, the main nutrients in the culture medium were analysed in the chemostat effluent, and the corresponding results are presented in Figure 4A–C. As shown in Figure 4A,B, nitrates and phosphates are the two nutrients that exhibit the greatest variation with increasing dilution rates, with nitrates playing a greater role and potentially becoming limiting at dilution rates below 0.3 d^−1^ under the culture medium and conditions used here. Higher dilution rates led to higher residual nutrient concentrations in the chemostat effluent, indicating that the microalgal culture left a larger fraction of these nutrients unconsumed under shorter reactor retention times. The consumption of both nitrate and phosphate nutrients decreased linearly with increasing dilution rate. At a dilution rate of 0.30 d^−1^, nearly 100% of the nitrates were consumed, whereas only about 24% of the phosphates were taken up by the microalgal culture. At a dilution rate of 1.00 d^−1^, the consumption of both these nutrients was nearly undetectable in the effluent. However, the BBM is phosphate-buffered, resulting in concentrations far exceeding the culture’s uptake need under the conditions used.

For sulfates (Figure 4A) and magnesium (Figure 4B), although they showed less pronounced variations than the primary nutrients (N and P sources), their residual concentrations in the effluent also tended to increase with higher dilution rates. At a dilution rate of 0.30 d^−1^, only 22% of the initial sulfate and 34% of the initial magnesium were consumed. At the highest dilution rate (1.00 d^−1^), their consumption was virtually undetectable. Chloride, sodium, potassium, and calcium, although consumed at basal levels, did not exhibit a clear trend of variation with increasing dilution rate.

Figure 4C presents the daily uptake (i.e., UR = uptake rate) of the two main macronutrients, nitrate (N-source) and phosphate (P-source) (inferred from their residual concentrations in the chemostat effluent), as well as the specific uptake rates (SUR) of these nutrients by the biomass in the reactor at each steady-state across the different dilution rates. As shown and considering that at a dilution rate of 0.30 d^−1^ virtually all the nitrate in the culture medium was consumed (Figure 4A), daily nitrate uptake increased from 281 mg/d at 0.30 d^−1^ to 318 mg/d at 0.40 d^−1^. Beyond this point, it progressively declined with increasing dilution rates, becoming nearly undetectable at a dilution rate of 1.00 d^−1^. This pattern suggests that at a rate of 0.30 d^−1^, the culture experienced nitrate limitation, leading to its complete consumption. At a rate of 0.40 d^−1^, the increased inflow of fresh medium supplied additional nitrate, allowing for higher uptake. However, as the dilution rate continued to rise and cells exceeded their optimal growth rate, nitrates assimilation efficiency decreased accordingly. Considering that biomass concentration decreased with increasing dilution rate from 0.30 d^−1^ onward (Figure 2), Figure 4C shows that the specific nitrate assimilation rate increased up to 112 mg/d/g at a 0.50 d^−1^ dilution rate. This trend may be attributed not only to the greater nitrate availability resulting from the higher inflow of fresh medium but also to the reduced biomass density, which likely enhanced light penetration and CO_2_ availability for the cells at each steady-state as the dilution rate increased. For phosphates, although not a limiting nutrient under the conditions tested, a similar trend was observed: specific uptake increased up to 21 mg/d/g at the dilution rate of 0.50 d^−1^ and then declined with further increases in dilution rate. These observations are consistent with findings in another microalga, *Chlorella vulgaris*, where macronutrient uptake was shown to depend not only on nutrient availability but also on cultivation conditions, including CO_2_ supply and the irradiance accessible to cells for photosynthesis [50].

The residual nutrient levels in the chemostat effluent clearly indicate that, at dilution rates above the optimal growth range (0.3–0.4 d^−1^) for *H. pluvialis*, nutrient consumption is suboptimal under the tested conditions, as the microalgal cells are subject to dilution rates too high for them to efficiently utilize the incoming nutrients before being washed out. This highlights the need to optimize the culture medium according to the cultivation conditions to minimize nutrient waste and enhance the sustainability of the microalgae biotechnological process that, although promising, remains economically challenging, as mentioned by Figueroa-Torres et al. [48]. Beyond medium optimization, nutrient recycling is essential to enhance productivity and improve process profitability [51]. To this purpose, strategies such as reusing the culture medium after microalgal harvesting and/or integrating it into sequential systems, where controlled nutrient deficiency is applied to stimulate the targeted production of metabolites of biotechnological interest, can play a key role in minimizing nutrient waste and mitigating economic and environmental impacts.

### 3.2. Chemostat and Sequential Columns—Configuration 2

#### 3.2.1. ST2 Operation in Continuous Mode

To enhance the CO_2_ absorption capacity of the PBR system and improve overall productivity, the system was operated in a second configuration consisting of a chemostat (stage 1) followed by a series of sequential columns (stage 2). In this configuration (configuration 2 = ST1 + ST2 PBR, Figure 1), while operated in continuous mode, the effluent culture of *H. pluvialis* and exhaust gases from the chemostat were directly channelled into the sequential columns, which were exposed to high irradiance (≈200 μmol·m^−2^·s^−1^).

**Figure 5:**
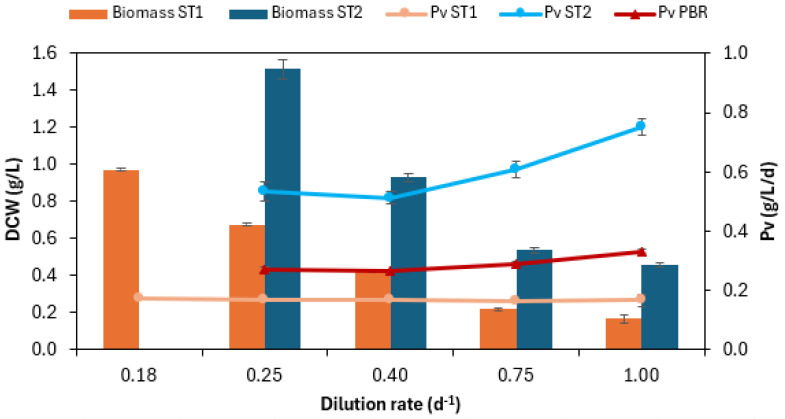
*H. pluvialis* biomass concentration (DCW) and productivity (Pv) in a two-stage PBR system, with stage 1 (ST1) operated as a chemostat at different dilution rates (0.18–1.0 d^−1^) and stage 2 (ST2) consisting of a sequential series of columns (i.e., Configuration 2 = ST1 + ST2 PBR). Error bars indicate the standard deviation of triplicate measurements (*n* = 3) at each steady-state.

Figure 5 illustrates biomass production for ST1 and ST1 + ST2, as well as productivity for ST1, ST2 and ST1 + ST2, at 5 different dilution rates (0.18–1.0 d^−1^). Table 2 summarizes the main metabolic parameters for ST1 + ST2 across the different dilution rates. As can be observed in Figure 5 and Table 2, in both stages (ST1 and ST2), the biomass concentration decreased with increasing dilution rate. In ST1, biomass concentration declined exponentially (R^2^ = 0.99) from 0.97 ± 0.01 g/L at 0.18 d^−1^ to 0.17 ± 0.02 g/L at 1.0 d^−1^. Similarly, even with the inclusion of ST2, an exponential decrease was still observed (R^2^ = 0.98) from 1.52 ± 0.05 g/L at 0.25 d^−1^ to 0.46 ± 0.01 g/L at 1.0 d^−1^. Nonetheless, at all dilution rates tested, with the addition of ST2 (configuration 2) the biomass concentration in the effluent culture (i.e., collected from the last column) was more than twice that obtained in ST1 effluent. Despite the decrease in biomass concentration in ST1 across dilution rates, volumetric productivity (Pv ST1—Figure 5) remained relatively stable at 0.169 ± 0.004 g/L/d between 0.18 and 1.0 d^−1^. Conversely, disregarding the contribution of ST1 and considering only the volume in ST2, volumetric productivity in this stage (Pv ST2—Figure 5) increased from 0.54 ± 0.04 g/L/d to 0.75 ± 0.03 g/L/d as the dilution rate increased from 0.25 to 1.0 day^−1^, despite the decrease in biomass concentration. Considering the overall two-stage PBR system (ST1 + ST2), the combined volumetric productivity (Pv PBR—Figure 5) increased from 0.267 ± 0.006 to 0.332 ± 0.008 g/L/d as the dilution rate increased from 0.4 to 1.0 d^−1^. Although ST2 only accounted for 28% of reactor volume, its inclusion in the combined two-stage PBR system, enabled higher biomass concentrations in the effluent *H. pluvialis* culture at all tested dilution rates (Figure S1, from Supplementary Materials) and also resulted in productivities 57% (at 0.4 d^−1^) to 99% (at 1.0 d^−1^) higher than those obtained in ST1 only. The maximum productivity in configuration 2 (0.332 ± 0.008 g/L/d at a dilution rate of 1.0 d^−1^) was relatively close to the maximum achieved in configuration 1, which consisted only of the chemostat stage (0.337 g/L/d at 0.3 day^−1^, see Table 1), corresponding to daily productivities of 3.65 g/d (Table 2) and 2.66 g/d (Table 1), respectively, for configuration 2 and configuration 1.

The results of this study highlight the critical role of light availability in enhancing the vegetative productivity of green cells in *H. pluvialis*. Previous studies have reported irradiance levels of 50–60 μmol photons·m^−2^·s^−1^ as optimal for vegetative growth [24,52,53,54], while other authors have shown that higher irradiance, up to 150 μmol photons·m^−2^·s^−1^, does not impair vegetative growth and may even enhance it [36,53,55]. Despite these differences, it is well-established that elevated irradiance is a key trigger for carotenoid, particularly astaxanthin, accumulation in *H. pluvialis*. High light acts as a stress factor that induces astaxanthin synthesis [36,56] and up-regulates essential carotenogenic genes under both light and nutrient stress conditions [57,58,59]. Due to the complex life cycle of *H. pluvialis* and its sensitivity to cultivation factors, this microalga is typically cultivated in two distinct stages: the first under mild, nutrient-replete conditions to promote rapid cell division and high cell density, and the second under stress conditions, mainly nitrogen limitation and high irradiance, to induce cell maturation and pigment accumulation, usually accompanied by growth arrest and encystment [22,54,60].

In this study, a two-stage continuous cultivation strategy was employed, with higher irradiance (≈200 μmol·m^−2^·s^−1^) applied during the second stage (ST2) to promote green cell maturation, biomass accumulation, and enhance CO_2_ capture efficiency in the biorefinery system. While industrial-scale cultivation of *H. pluvialis* typically involves a first stage of vegetative growth followed by a second batch stage for carotenogenesis induction [22,60], the two-stage strategy herein was primarily intended to evaluate the possibility of extending biomass accumulation in continuous mode before the batch step. It was observed that, in addition to the increase in biomass concentration, cell density also increased during the second stage, suggesting that under these continuous culture conditions, the cells either did not experience sufficient stress to trigger division arrest or did not remain in the second stage long enough at these dilution rates to initiate carotenogenesis.

#### 3.2.2. ST2 Operation in Batch Mode

Considering the previous findings, and to evaluate the induction of carotenogenesis, two further assays were performed using the ST2 in batch mode, with the chemostat maintained at a dilution rate of 0.50 d^−1^. As mentioned in a previous section (Section 2.3.2), for these assays, culture transfer from ST1 to ST2 was interrupted, cells produced in ST1 were diverted towards a collecting vessel, and only the exhaust air from the ST1 was transferred to the ST2.

Operating under batch conditions, the green batch culture in ST2 initially consumed nearly all the CO_2_ from the supplied air. As the culture developed, CO_2_ consumption was progressively reduced, and, after 13 days, once CO_2_ assimilation stopped, the culture was harvested. During this 13-day carotenogenesis period, the culture gradually changed from green to a dense dark green and then to an orange tone, which became increasingly intense toward the end of the induction phase (Figure S2, from Supplementary Materials), reaching a final biomass concentration of 3.33 ± 0.16 g/L. The duration of carotenogenesis observed in this study is consistent with previous findings. For instance, Fábregas et al. [22] reported that inducing the transition to the aplanospore stage and astaxanthin accumulation required approximately 15 days, during which the culture continued to grow until all nitrogen was depleted. However, the duration of carotenogenesis varies considerably and is strongly influenced by nutritional conditions and irradiance levels, as demonstrated by Pereira & Otero [24]. In summary, these results indicate that the vegetative phase of *H. pluvialis* can be extended in continuous culture under increased irradiance without inducing growth arrest, thereby enhancing biomass concentration. However, under these conditions, carotenoid accumulation, essential for sustained astaxanthin production, is not triggered. So, to achieve this, it is necessary to subject the cells to a combined light and nutrient stress under conditions more typical of batch culture, allowing sufficient exposure time to induce the physiological and morphological changes necessary for maximal astaxanthin synthesis. Such conditions could likely be achieved by further increasing the volume of ST2 and reducing the overall dilution rates or reducing nutrient concentration in culture medium.

Over the last few years, efforts to shorten the carotenogenesis induction phase in *H. pluvialis* have focused on process optimization and environmental fine-tuning. The adjustment of light quality, spectrum, and temperature has been shown to promote astaxanthin accumulation by regulating key metabolic pathways [24,56]. Likewise, staged cultivation strategies combining moderate light stress, controlled nitrogen limitation, and supplementation with organic carbon sources have been reported to accelerate carotenoid induction while minimizing cell mortality [60]. More recently, modulation of nitrogen availability and irradiance during the motile-cell stage [59], as well as the use of biostimulants such as native exopolysaccharides [25], have shown potential to further enhance astaxanthin accumulation and process performance.

### 3.3. Profiles of CO_2_ Uptake and O_2_ Release: Configuration 1 vs. Configuration 2

#### 3.3.1. Single-Stage Chemostat PBR—Configuration 1 (ST1)

**Figure 6:**
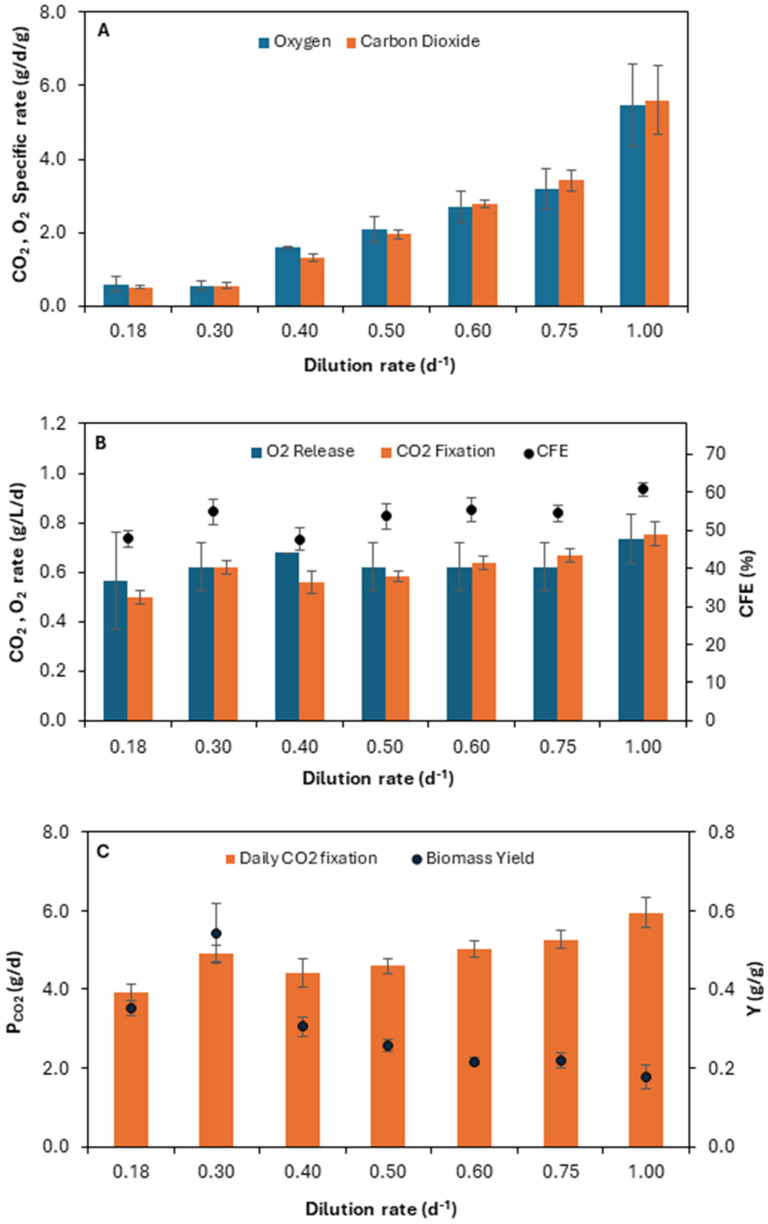
Dynamics of CO_2_ sequestration and O_2_ release by *H. pluvialis* as a function of dilution rate (0.18–1.00 d^−1^) in continuous culture within the single-stage PBR system (Configuration 1 = ST1 PBR). Plot (**A**) shows the specific rates (A_CO2_, A_O2_), plot (**B**) shows the volumetric rates, and plot (**C**) shows the daily CO_2_ fixation alongside the corresponding biomass yield (i.e., incorporation of fixed CO_2_ into biomass). Error bars represent the standard deviation of triplicate measurements (*n* = 3) at each steady-state.

The dynamics of CO_2_ uptake and O_2_ production in the chemostat (ST1 PBR) were evaluated at steady-state for each dilution rate (0.18–1.00 d^−1^), with the results presented in Figure 6A–C. As shown in Figure 6A, from 0.3 to 1.0 d^−1^, specific CO_2_ uptake (A_CO2_) and O_2_ production (A_O2_) increased linearly with dilution rate (R^2^ = 0.992 and R^2^ = 0.976, respectively). The specific CO_2_ fixation increased from 0.512 g/d/g at a dilution rate of 0.18 d^−1^ to 5.607 g/d/g at 1.00 d^−1^, while specific O_2_ production increased from 0.581 g/d/g to 5.464 g/d/g over the same range, corresponding to approximately 11-fold and 9-fold increases, respectively. These results indicate that as the dilution rate increased, the culture’s metabolic activity intensified. Shorter cell retention times at higher dilution rates promoted faster growth, which in turn led to higher rates of CO_2_ fixation and O_2_ production. It is also possible that, under constant and continuous illumination at higher dilution rates, the lower cell concentration reduces self-shading, thereby enhancing photosynthetic activity within the microalgal population. Fierro et al. [61] have suggested that this feature can be exploited to enhance productivity in continuous photobioreactors.

Figure 6B shows the CO_2_ assimilation capacity (CO_2_ AC = CO_2_ rate) and O_2_ production capacity (O_2_ PC = O_2_ rate) of *H. pluvialis* at each steady-state across the tested dilution rates (0.18–1.00 d^−1^). Except for the peak at 0.30 d^−1^, the CO_2_ AC increased consistently with dilution rate, ranging from 0.497 g/L/d at 0.18 d^−1^ to 0.753 g/L/d at 1.00 d^−1^. In contrast, the O_2_ PC showed a general increasing trend with dilution rate, although the variability observed rendered these differences not significant. The maximum CO_2_ AC achieved in this single-stage configuration study (0.753 g/L/d) represents a 32.6% improvement compared to the maximum value (0.568 g/L/d) previously reported by Tavares et al. [21] for this microalgae under the same type of photobioreactor. These values are consistent with those generally reported for the CO_2_ assimilation capacity of *H. pluvialis* [62] at CO_2_ concentrations of up to 5 vol%. However, CO_2_ fixation by microalgae can vary depending on the concentration and type of available carbon sources [63]. Specifically, CO_2_ concentration significantly influences microalgal growth, enhancing growth up to an optimal level, beyond which excess CO_2_ can become inhibitory if culture conditions are not well controlled [64,65]. Considering the low CO_2_ influx of 0.29 ± 0.04 vol% applied in this study, the observed CO_2_ assimilation capacity reflects the strong photosynthetic performance of the culture within the continuous PBR system. Furthermore, as shown in Figure 6B and Table 1, the *H. pluvialis* culture in this chemostat PBR system exhibited a carbon fixation efficiency (CFE) ranging from 47.6% to 60.7% across the different dilution rates. Regarding daily CO_2_ fixation (P_CO2_) (Table 1), values increased progressively with higher dilution rates, except at 0.30 d^−1^, where a peak was observed. The P_CO2_ increased from 3.92 g/d at 0.18 d^−1^ to 5.95 g/d at the highest dilution rate tested (1.00 d^−1^). However, as shown in Figure 6C, this increase in P_CO2_ did not translate proportionally into biomass incorporation beyond the dilution rate of 0.30 d^−1^. From this point onwards, the biomass yield (Y, g/g) per unit of fixed CO_2_ declined as the dilution rate increased. Since biomass volumetric productivity (Pv) remained at a plateau (≈0.14 g/L/d) for dilution rates above 0.30 d^−1^ (Figure 2), the additional fixed CO_2_ at higher dilution rates appears to be redirected away from structural biomass synthesis. This observation suggests under-utilization of the available light energy and insufficient nutrient assimilation, whereby fixed carbon is not efficiently converted into cellular biomass due to intracellular metabolic bottlenecks rather than active biomass accumulation. Such behavior is consistent with previous reports indicating that, under these potentially stress-inducing conditions, a fraction of the fixed CO_2_ may be diverted toward the synthesis of secondary metabolites that are subsequently released into the culture medium or lost as biogenic volatile organic compounds through the exhaust gases [25,66].

#### 3.3.2. Two-Stage PBR System—Configuration 2 (ST1 + ST2)

**Figure 7:**
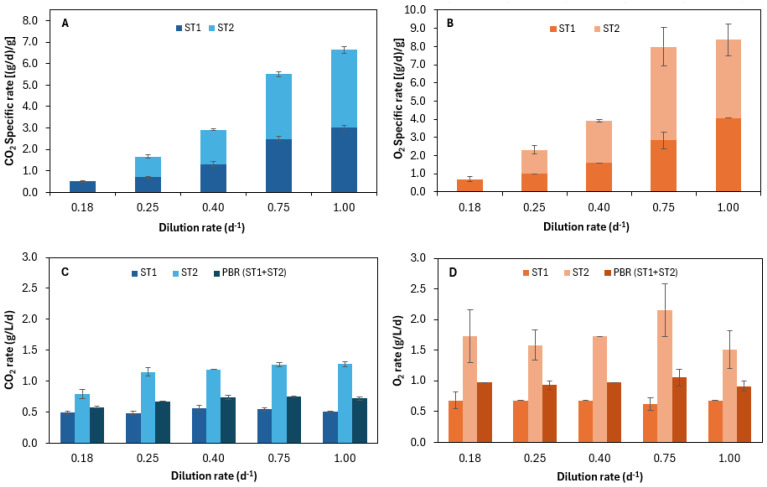
Dynamics of CO_2_ sequestration and O_2_ release by *H. pluvialis* as a function of dilution rate (0.18–1.00 d^−1^) in continuous culture within the two-stage PBR system (Configuration 2 = ST1 + ST2 PBR). Plots (**A**,**B**) show specific rates (A_CO2_, A_O2_), while plots (**C**,**D**) show volumetric rates (CO_2_ rate = CO_2_ AC and O_2_ rate = O2 PC). Error bars represent the standard deviation of triplicate measurements (*n* = 3) at each steady-state.

Considering the goals of enhancing CO_2_ capture efficiency in the two-stage PBR system (Configuration 2—ST1 + ST2), the kinetics of CO_2_ fixation and O_2_ release were analysed, and the results are presented in Figure 7A–D. As shown in Figure 7A,B, the specific rates of CO_2_ assimilation (A_CO2_) and O_2_ production (A_O2_) increased with higher dilution rates in ST1, consistent with the trend observed in configuration 1 of the PBR system. This behaviour may reflect an increase in metabolic activity resulting from the cells being forced to grow at an increasing dilution rate above their typical growth range. The observed CO_2_ specific rates were also slightly higher in ST2 than in ST1, which was to be expected and, as mentioned above, may be explained by the greater light exposure in ST2, which would result in greater energy availability and increased photosynthetic activity. Overall, the results indicate that, at the scale of the whole PBR system (ST1 + ST2 PBR system = configuration 2), metabolic activity increased with dilution rate, as each unit of biomass exhibited higher specific CO_2_ fixation and O_2_ release at elevated dilution rates.

Considering the analysis of CO_2_ assimilation capacity (CO_2_ AC = CO_2_ rate) and O_2_ production capacity (O_2_ PC = O_2_ rate) in each PBR stage individually (Figure 7C,D), we observe that in ST1 both rates remain relatively stable across the different dilution rates, with CO_2_ ranging from 0.481 to 0.559 g/L/d and O_2_ from 0.621 to 0.677 g/L/d. However, when analysing ST2 alone, the CO_2_ assimilation capacity increased markedly compared to ST1 and with rising dilution rates, from 0.791 g/L/d at 0.18 d^−1^ dilution rate to 1.275 g/L/d at 1.0 d^−1^ dilution rate. In contrast, values for O_2_ production capacity in ST2 ranged from a minimum of 1.510 g/L/d (1.0 d^−1^) to a maximum of 2.157 g/L/d (0.75 d^−1^), but no clear and significant trend was observed due to the variance of this parameter. These data are closely aligned with the observations described in Section 3.2, indicating that the improved performance in CO_2_ assimilation and O_2_ production in ST2 is linked to the enhanced vegetative productivity of *H. pluvialis* green cells resulting from the high irradiance and light availability applied in the second stage of the PBR system.

Regarding the CO_2_ assimilation capacity (CO_2_ AC = CO_2_ rate) of the entire PBR system (ST1 + ST2), shown in Table 2 and Figure 7C, it increased from 0.580 g/L/d at the lowest dilution rate (0.18 d^−1^) to 0.747 g/L/d at 0.75 d^−1^, before decreasing slightly to 0.719 g/L/d at the highest dilution rate tested (1.00 d^−1^). As for the oxygen production capacity (O_2_ PC = O_2_ rate) (Table 2 and Figure 7D), the values obtained ranged from 0.912 g/L/d at the highest dilution rate (1.00 d^−1^) to 1.053 g/L/d at 0.75 d^−1^. Despite this variation, the observed fluctuations do not reveal a clear or significant trend. The maximum CO_2_ AC (0.747 g/L/d) achieved in this two-stage PBR system is significantly higher than the maximum CO_2_ AC of 0.613 g/L/d reported by Azizi et al. [62], when employed a CO_2_ steady-feeding strategy based on pH stability under high light intensities (450 µmol photons/m^2^/s). This result may be attributed to the fact that sequential photobioreactors, such as the configuration used in this study, enhance CO_2_ assimilation by ensuring a more continuous and efficient supply of CO_2_ to the microalgae. This sequential configuration (ST1 + ST2 PBR) helps prevent CO_2_ depletion in the initial stage and improves the overall conversion efficiency, as reported by Lee et al. [38]. However, it should be noted, from Figure 7C,D, that the ratio between O_2_ produced and CO_2_ consumed exceeds 1 (1.3–1.7), deviating from the ideal stoichiometric balance of 1:1. This deviation may be associated with O_2_ accumulation, which is a typical limitation in sequential photobioreactors and can partially inhibit photosynthesis [67]. Consequently, further effective management of dissolved oxygen levels is essential to sustain high photosynthetic efficiency [68].

**Figure 8:**
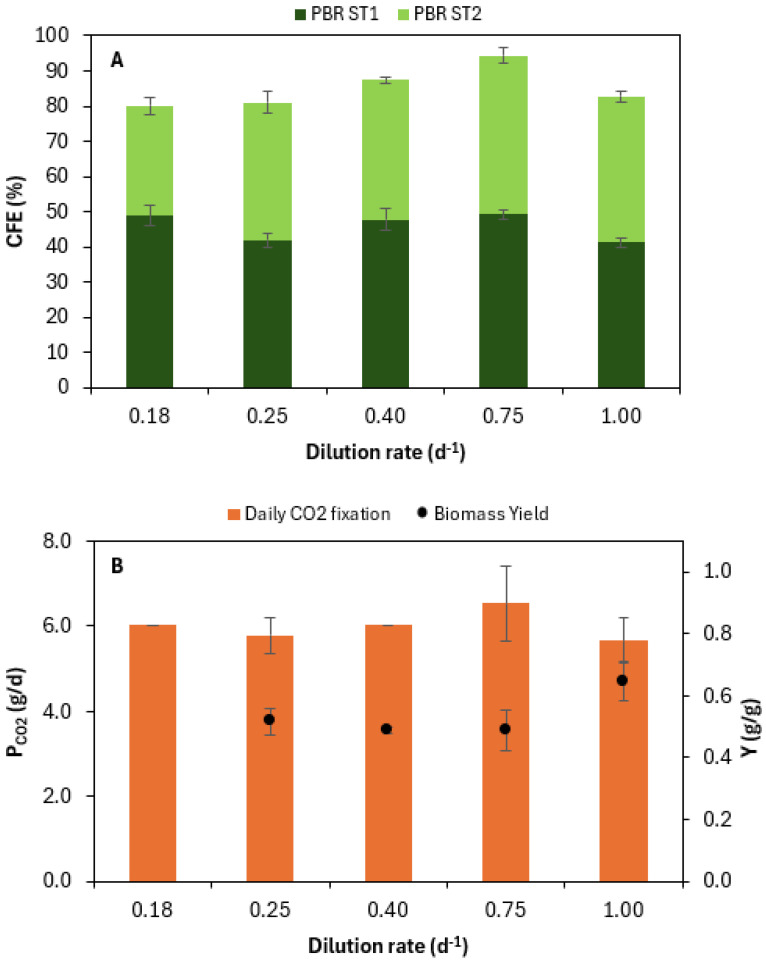
Carbon fixation performance of *H. pluvialis* in the two-stage PBR system (Configuration 2 = ST1 + ST2 PBR) across different dilution rates (0.18–1.00 d^−1^). The plot (**A**) shows CO_2_ fixation efficiency (CFE), while the plot (**B**) presents daily CO_2_ fixation (P_CO2_) and the corresponding biomass yield (Y). Error bars represent the standard deviation of triplicate measurements (*n* = 3) at each steady-state.

Considering that the primary goal of implementing a second stage in the upgraded chemostat PBR system was to enhance biogenic CO_2_ fixation efficiency (CFE), Figure 8A illustrates the CFE profiles across the tested dilution rates for ST1 and ST2 within configuration 2. As shown, CFE increased progressively from 80% at the lowest dilution rate (0.18 d^−1^) to a maximum of 94% at 0.75 d^−1^, before declining again to 83% at the highest dilution rate (1.00 d^−1^). Despite the CO_2_ influx of 0.29 ± 0.04 vol% applied in this study, the observed CO_2_ AC and the CFE reflect the strong photosynthetic performance of the *H. pluvialis* culture within the continuous PBR system and show how sequential photobioreactor systems can effectively improve CO_2_ fixation efficiency, thereby minimizing its release into the atmosphere. Figure 8B shows that daily CO_2_ fixation (P_CO2_) in the whole two-stage PBR system (configuration 2 = ST1 + ST2 PBR) remained relatively stable at 6.0 ± 0.3 g/d across the different dilution rates. This P_CO2_ value attained represents a 27.7% improvement compared to the average value (4.7 ± 0.08 g/d) achieved in the single-stage configuration (Configuration 1 = ST1 PBR). Moreover, the P_CO2_ obtained here is almost double the value (3.4 ± 0.1 g/d) previously reported by Tavares et al. [21] using the same microalga species and an identical photobioreactor design, which demonstrates the effectiveness of the improvements introduced in the system with the adoption of the second sequential stage. Relatively to the biomass yield per unit of fixed CO_2_ (Y), it remained relatively constant from 0.25 d^−1^ to 0.75 d^−1^ (0.47 g/g and 0.48 g/g, respectively), and then increased to 0.59 g/g at the highest dilution rate tested (1.00 d^−1^). The maximum value obtained here is higher than the peak biomass yield per unit of fixed CO_2_ (0.54 g/g at a dilution rate of 0.30 d^−1^) observed in the single-stage configuration, and exceeds all the yields measured at the other dilution rates tested in that configuration (Table 1). This suggests that single-stage conditions may favor the incorporation of fixed CO_2_ into biomass only at the optimal growth rate for *H. pluvialis*. However, as shown in Figure 5, the volumetric productivity of the PBR system in configuration 2 (Pv PBR) exceeds that of ST1 alone (Pv ST1) and matches the maximum value achieved in the single-stage configuration (Pv in Configuration 1—Table 1). It should be noted that the ratio between cell biomass produced and fixed CO_2_ may underestimate total carbon assimilation, as part of the fixed carbon can be redirected toward extracellular or volatile metabolites [25,66,69,70].

By enabling the enhanced capture and biological utilization of biogenic CO_2_ streams that would otherwise be released into the atmosphere, the proposed two-stage photobioreactor can make an important contribution to carbon neutrality efforts. The microalgal biomass produced from this integration can serve as the basis for the production of low carbon sustainable fuels, such sustainable aviation fuels, as well as the procution of low emission food and feed alternatives, promoting the valorization of waste carbon within a circular biorefinery framework, simultaneously reducing emissions and generating value-added products [71,72,73]. These environmental and economic benefits highlight the potential of this approach to support more sustainable industrial processes.

## 4. Conclusions

In this study, an upgraded version of a photobioreactor (PBR) was developed for continuous microalgae cultivation: a two-stage system (ST1 + ST2 PBR) composed of a chemostat (ST1) followed by sequential illuminated columns (ST2). This system was designed to improve CO_2_ absorption, to extend biomass accumulation under continuous operation, and to stimulate the carotenogenesis step.

Due to its well-known industrial relevance, *H. pluvialis* was used as the model strain. The results obtained across the different dilution rates (0.18–1.0 d^−1^) demonstrated the strong potential of this PBR system for improved productivity and CO_2_ capture. In the single-stage configuration (ST1 PBR), the peak volumetric productivity was 0.337 g/L/d at 0.30 d^−1^, with a daily productivity of 2.66 g/d. The two-stage system (ST1 + ST2 PBR) enabled higher biomass concentrations in the ST2 effluent culture at all dilution rates and resulted in a maximum overall volumetric productivity of 0.332 g/L/d and a daily productivity of 3.65 g/d at the highest dilution rate (1.0 d^−1^). In the single-stage configuration (ST1 PBR), the maximum CO_2_ assimilation capacity (AC) of 0.753 g/L/d and the highest CO_2_ fixation efficiency (CFE) of 60.7% were achieved at a dilution rate of 1.0 d^−1^, leading to a maximum daily CO_2_ fixation of 5.95 g/d. In the two-stage system (ST1 + ST2 PBR), the maximum AC of 0.747 g/L/d, CFE of 94%, and daily CO_2_ fixation of 6.53 g/d were all obtained at 0.75 d^−1^.

These results, together with the enhanced metabolic activity, highlight the effectiveness of the upgraded system in maximizing carbon utilization from low-concentration biogenic CO_2_ streams, while maintaining high productivity and operational stability across a broad range of dilution rates. This two-stage PBR system enabled the use of residual nutrients from the first stage to boost biomass productivity in the second. By recovering unconsumed nutrients, the system minimized nutrient waste and effluent treatment requirements, enhancing overall sustainability and economic viability. However, the continuous cultivation mode did not trigger carotenogenesis in the second stage under the tested light conditions (≈200 μmol·m^−2^·s^−1^), which only occurred under batch conditions where combined nutrient stress and prolonged high illumination induced the process. Overall, the ST1 + ST2 PBR system demonstrated high efficiency in CO_2_ capture, nutrient utilization, and biomass production, confirming its potential as a scalable and sustainable platform for microalgal cultivation. The system’s ability to couple heterotrophic CO_2_ reuse with continuous autotrophic growth highlights its relevance for industrial biorefineries and carbon mitigation strategies, contributing to the advancement of circular bioeconomy approaches.

## Figures and Tables

**Table 1:** Kinetic parameters of *H. pluvialis* growth in a continuous chemostat PBR (Configuration 1 = ST1 PBR, Figure 1) measured at steady states across the tested dilution rates (0.18–1.0 d^−1^).

Kinetic Parameters	Dilution Rates (d^−1^)						
	0.18	0.30	0.40	0.50	0.60	0.75	1.00
DCW (g/L)	0.97 (±0.01)	1.12 (±0.15)	0.43 (±0.01)	0.30 (±0.01)	0.23 (±0.00)	0.20 (±0.02)	0.13 (±0.02)
Px (g/d)	1.38 (±0.01)	2.66 (±0.35)	1.35 (±0.02)	1.18 (±0.05)	1.09 (±0.01)	1.16 (±0.09)	1.06 (±0.17)
Pv (g/L/d)	0.175 (±0.00)	0.337 (±0.04)	0.171 (±0.00)	0.150 (±0.01)	0.138 (±0.00)	0.146 (±0.01)	0.134 (±0.02)
AC (g/L/d)	0.497 (±0.03)	0.621 (±0.03)	0.559 (±0.05)	0.582 (±0.02)	0.636 (±0.03)	0.667 (±0.03)	0.753 (±0.05)
PC (g/L/d)	0.564 (±0.00)	0.621 (±0.10)	0.677 (±0.00)	0.621 (±0.10)	0.621 (±0.10)	0.621 (±0.10)	0.733 (±0.10)
P_CO2_ (g/d)	3.92 (±0.21)	4.90 (±0.21)	4.41 (±0.37)	4.60 (±0.18)	5.03 (±0.21)	5.27 (±0.21)	5.95 (±0.38)
CFE (%)	47.8 (±2)	54.8 (±3)	47.6 (±3)	53.6 (±3)	55.4 (±3)	54.5 (±2)	60.7 (±2)
Y (g/g)	0.35 (±0.02)	0.54 (±0.07)	0.31 (±0.03)	0.26 (±0.02)	0.22 (±0.01)	0.22 (±0.02)	0.18 (±0.03)

Abbreviations: DCW—dry cell weight of biomass; Px—biomass productivity; Pv—volumetric productivity; AC—CO_2_ assimilation capacity; PC—daily O_2_ production capacity; P_CO2_—daily CO_2_ fixation; CFE—carbon fixation efficiency; Y—biomass yield (biomass/CO_2_). Kinetic parameter definitions and calculation procedures were adopted from Tavares et al. [21]. The standard deviation (SD) is shown in parentheses. SD of 0.00 reflect the 0.1% O_2_ resolution limit of the measurement device.

**Table 2:** Kinetic parameters of *H. pluvialis* growth in a two-stage continuous PBR (Configuration 2 = ST1 + ST2 PBR, Figure 1) measured at steady states across the tested dilution rates (0.18–1.0 d^−1^).

Kinetic Parameters	Dilution Rates (d^−1^)				
	0.18 ^a^	0.25 ^a^	0.4 ^a^	0.75 ^a^	1.00 ^a^
DCW (g/L) ^b^	ND ^c^	1.52	0.93	0.54	0.46
		(±0.05)	(±0.02)	(±0.01)	(±0.01)
Px (g/d)	ND	2.99	2.94	3.19	3.65
		(±0.10)	(±0.06)	(±0.09)	(±0.09)
Pv (g/L/d)	ND	0.27	0.27	0.29	0.33
		(±0.01)	(±0.01)	(±0.01)	(±0.01)
AC (g/L/d)	0.580	0.669	0.736	0.747	0.719
	(±0.019)	(±0.00)	(±0.033)	(±0.010)	(±0.024)
PC (g/L/d)	0.972	0.932	0.972	1.053	0.912
	(±0.000)	(±0.070)	(±0.000)	(±0.140)	(±0.086)
P_CO2_ (g/d)	6.03	8.78	6.03	6.53	6.65
	(±0.00)	(±0.44)	(±0.00)	(±0.87)	(±0.53)
CFE (%)	80.1	81.1	87.4	94.4	82.7
	(±3.9)	(±3.5)	(±3.2)	(±2.7)	(±1.9)
Y (g/g)	ND	0.52	0.49	0.49	0.65
		(±0.04)	(±0.01)	(±0.07)	(±0.06)

Abbreviations: DCW—dry cell weight of biomass; Px—biomass productivity; Pv—volumetric productivity; AC—CO_2_ assimilation capacity; PC—daily O_2_ production capacity; P_CO2_—daily CO_2_ fixation; CFE—carbon fixation efficiency; Y—biomass yield (biomass/CO_2_). The standard deviation is shown in parentheses. Notes: ^a^ Reference dilution rate corresponding to stage 1 (ST1); ^b^ Concentration determined in the ST2 culture effluent (collected from the last column). ^c^ ND—not determined (insufficient sample).

## Data Availability

The authors confirm that the datasets supporting the findings and conclusions of this study are available within the article.

## Supplementary Materials

The following supporting information can be downloaded at https://www.mdpi.com/article/10.3390/molecules31152627/s1, Figure S1. Biomass production at similar dilution rates within ST1 and ST1+2 configurations; Figure S2. *H. pluvialis* cultured under carotenogenic conditions.
