# Supplementary material for: Enhanced CO2 Fixation Through Continuous Cultivation of Microalgae in a Two-Stage Photobioreactor System
Source: Molecules. 2026 Jul 28;31(15):2627. doi: 10.3390/molecules31152627 (PMC13468652; doi:10.3390/molecules31152627)
Supplement: Supplementary file 1 [file molecules-31-02627-s001.zip › molecules-4374563-supplementary.pdf]

Supplementary for

Enhanced CO_2 Fixation Through Continuous Cultivation of Microalgae in a Two-Stage Photobioreactor System

João Tavares *, Susana M. Paixão *, Tiago P. Silva † and Luís Alves *

Unidade de Bioenergia e Biorrefinarias, LNEG—Laboratorio Nacional de Energia e Geologia I.P., Estrada do Paço do Lumiar 22, 1649-038 Lisboa, Portugal

* Correspondence: joao.tavares@lneg.pt (J.T.); susana.alves@lneg.pt (S.M.P.); luis.alves@lneg.pt (L.A.)

† Current address: ApC—Agência para o Clima I.P., Rua de "O Século", n.º 51/63, 1200-433 Lisboa, Portugal.

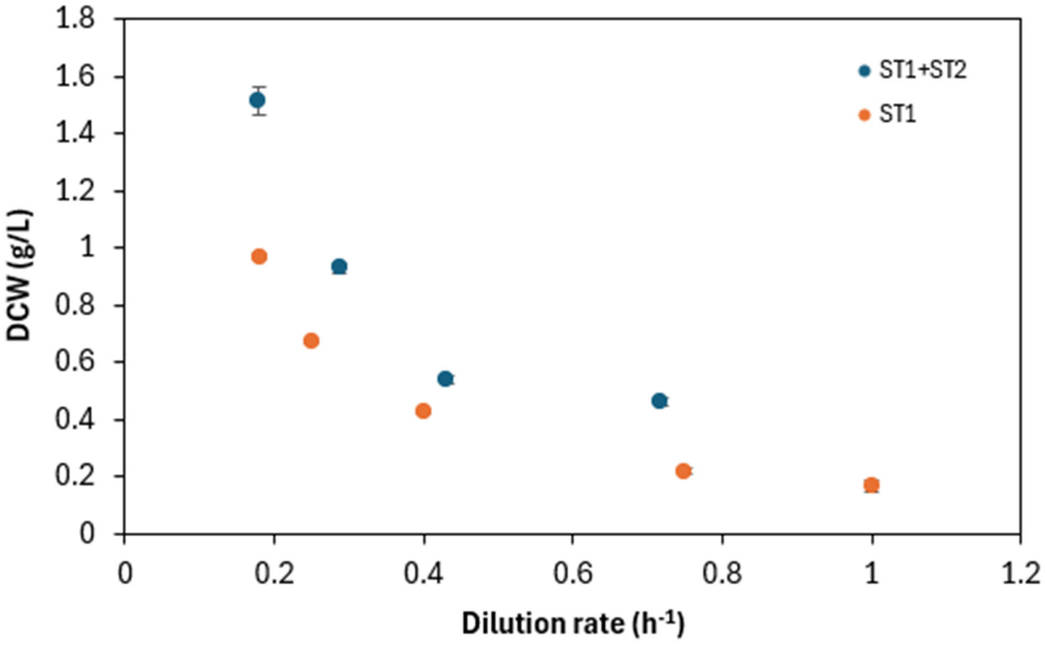

Figure S1. Biomass concentration (DCW, g/L) in the effluent of ST1 and ST1+ST2 configurations, at different dilution rates, adjusting the dilution rate for ST1+ST2, for the values considering the complete system volume.

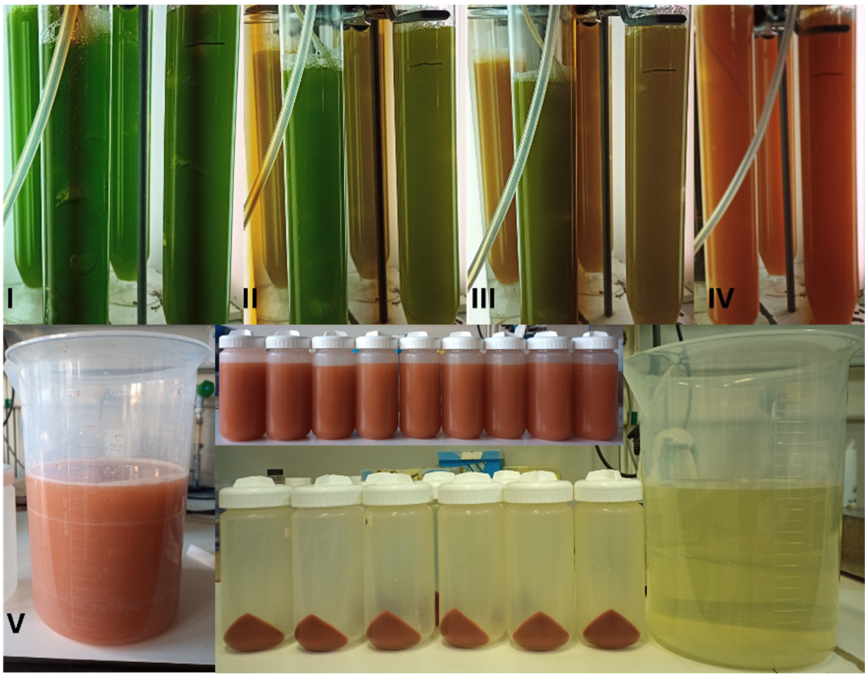

Figure S2. *H. pluvialis* cultured under carotenogenic conditions. I – initiation of carotenogenesis in batch culture; II – 5 days after induction; III – 8 days after induction; IV – 13 days after induction; V – biomass harvesting and separation.
